# Surgical Explantation of Impella 5.5 With Inflow Thrombus in Patients Undergoing Durable Left Ventricular Assist Device Implantation

**DOI:** 10.1177/15569845231196862

**Published:** 2023-09-14

**Authors:** Ismael A. Salas De Armas, Juan Marcano, Mehmet H. Akay, Manish K. Patel, Jayeshkumar Patel, Dina Al Rameni, Biswajit Kar, Igor D. Gregoric

**Affiliations:** 1 Department of Advanced Cardiopulmonary Therapies and Transplantation, The University of Texas Health Science Center at Houston, TX, USA

**Keywords:** heart failure, mechanical circulatory support, Impella, thrombus, left ventricular assist device

## Abstract

The Impella 5.5^®^ (Abiomed, Danvers, MA, USA) is a microaxial flow pump that promotes left ventricular unloading and improves end-organ perfusion before durable left ventricular assist device (LVAD) implantation. Thrombus formation after Impella 5.5 insertion can occur and represents a significant challenge to device explantation. Durable LVAD implantation is typically performed without aortic cross-clamping, so a dislodged thrombus can potentially embolize and lead to catastrophic events. We describe our technique to safely explant an Impella 5.5 in patients who develop a thrombus on the inflow portion of the device before surgical LVAD implantation.

## Graphical abstract

**Visual abstract:**
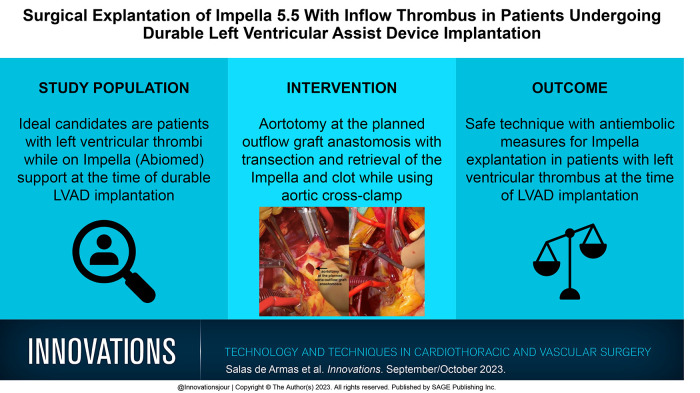

**Central Message**

Our technique highlights a practical solution to minimize the risk of embolization in patients with left ventricular thrombus at the time of switching from Impella to durable LVAD support.

## Introduction

The Impella 5.5^®^ device (Abiomed, Danvers, MA, USA) is a microaxial pump that shunts blood from the left ventricle (LV) to the aorta; it improves LV unloading in patients with cardiogenic shock.^1^ In the United States, the use of Impella devices is increasing for multiple indications.^2,3^ An LV thrombus is a contraindication for inserting any Impella device.^4^ However, thrombus formation can occur after Impella support is initiated by a combination of profound impairment of LV contractility, a poor flow state at the time of Impella 5.5 insertion, turbulent flow in the LV, or the thrombogenic nature of the device.^5 [Bibr bibr6-15569845231196862]^ A patient with an LV and/or inflow site thrombus presents a challenging dilemma, as the clot could lead to systemic emboli at the time of device explantation.

The removal of Impella devices is usually done by pulling the device out of the LV through the original insertion site (typically the axillary artery), followed by vascular repair of the access site. Device removal in the presence of an LV or inflow site thrombus could result in systemic embolization.^6^ There is little information regarding the surgical approach to managing a thrombus at the time of durable left ventricular assist device (LVAD) implantation. We describe our step-by-step technique of how to safely explant the Impella 5.5 in the presence of an inflow site thrombus in patients undergoing definitive surgical LVAD implantation.

## Surgical Technique

The patient in this report is a 62-year-old man with chronic systolic heart failure and a dilated ventricle with an end-diastolic diameter of 6.7 cm. He was transferred to our center with acute decompensation requiring dual inotropic support. In addition, he was deconditioned with impaired nutrition. The decision was to proceed with the implantation of the Impella 5.5 as a bridge to durable LVAD. The patient received Impella 5.5 support for 16 days. He was on systemic heparin with a partial thromboplastin time goal of 60 to 70 s; there was no evidence of hemolysis.

**Fig. 1.**
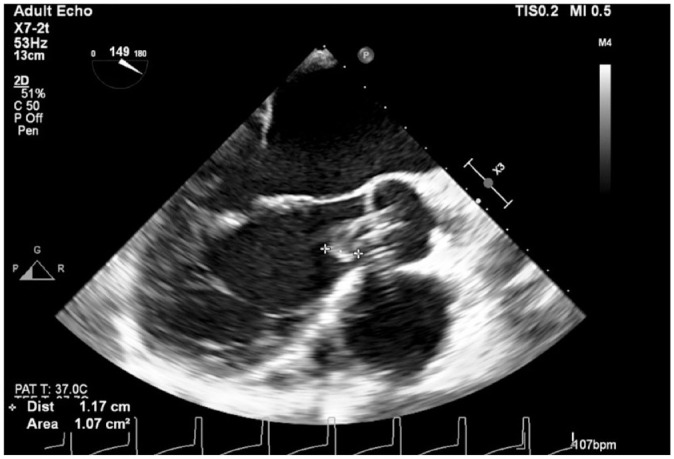
Transesophageal echocardiogram (long-axis aortic view) showing the Impella cannula (Abiomed, Danvers, MA, USA) in the left ventricle with a 1 cm clot on the cannula toward the inflow portion.

**Fig. 2.**
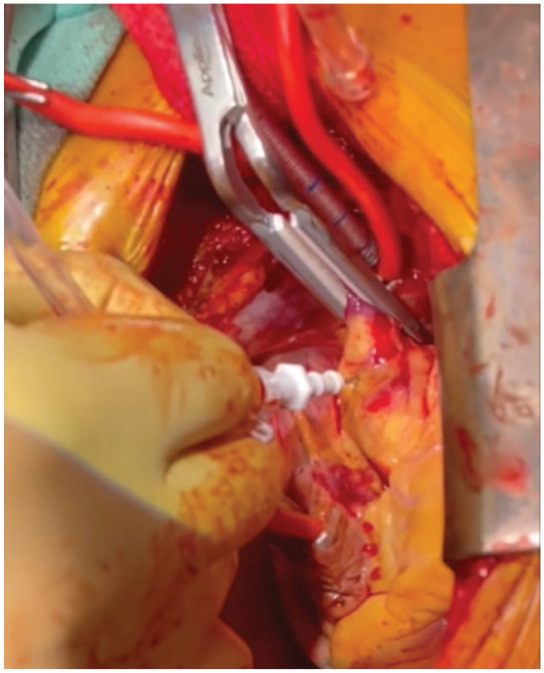
Cardiac diastolic arrest using paddle cross-clamp and antegrade cardioplegia needle.

The patient was taken to the operating room, placed in a supine position, and placed under general anesthesia. A transesophageal echocardiogram (TEE) probe was inserted before surgical incision to check the Impella device position, assess the presence of a thrombus, and determine the exact location and configuration of the clot (Fig. 1). A conventional median sternotomy was completed, and systemic heparin was given. Cannulas for cardiopulmonary bypass (CPB) support were placed in standard fashion. CPB was initiated, and the Impella flow was decreased. The CPB flow was increased while the Impella was turned off to avoid ventricular ejection. The padded aorta cross-clamp was placed across the aorta and the Impella catheter, and the heart was arrested using cardioplegia (Fig. 2).

**Fig. 3.**
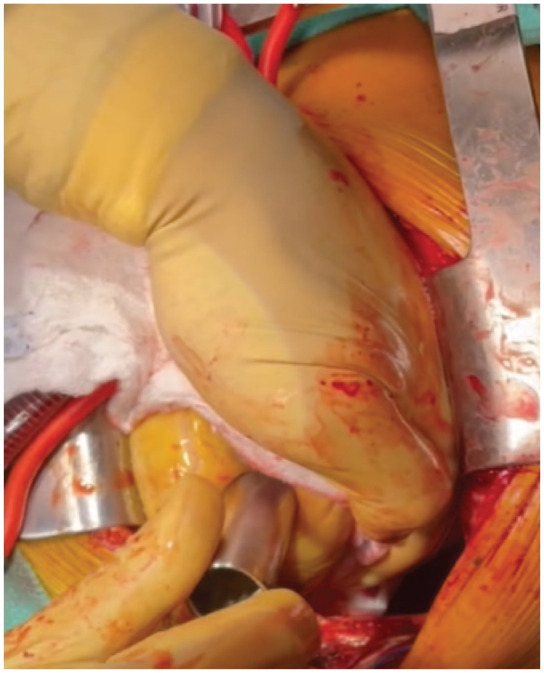
Left ventriculostomy with coring knife before suturing a sewing ring to allow better ventricular inspection.

**Fig. 4.**
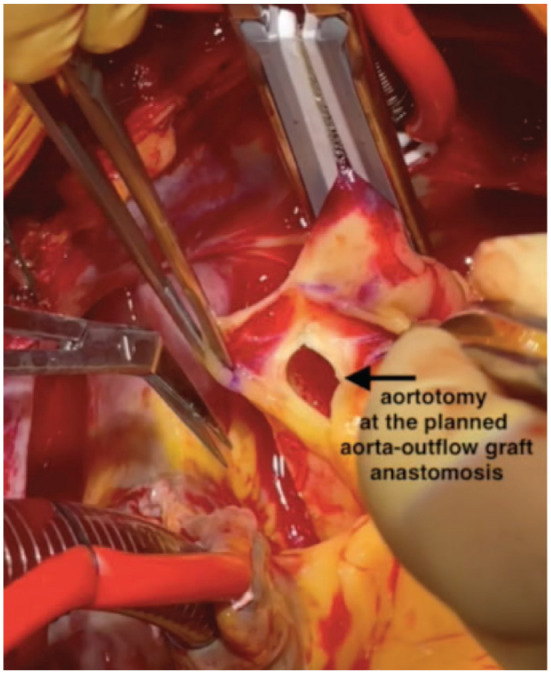
Aortotomy at the planned aorta-outflow graft anastomosis to expose the Impella (Abiomed, Danvers, MA, USA).

**Fig. 5.**
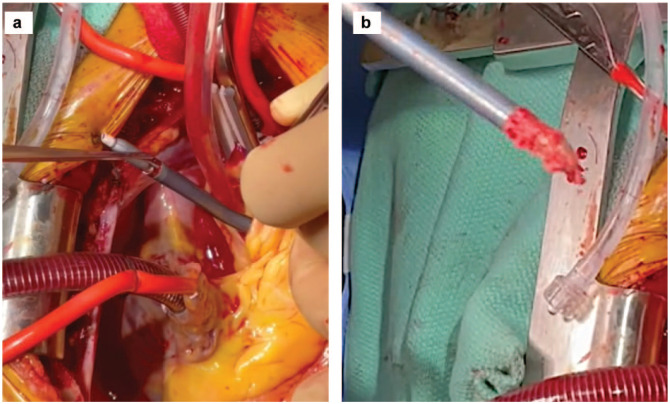
(a) Transaortic Impella (Abiomed, Danvers, MA, USA) transection and transaortic removal. (b) Impella removal with clot.

The heart was gently lifted, and a HeartMate 3 coring knife (Abbott Laboratories, Chicago, IL, USA) was used to perform a full-thickness apical left ventriculotomy (Fig. 3). A vertical aortotomy was made at the planned site for outflow graft anastomosis (Fig. 4). The Impella catheter was identified and transected using a wire cutter. The Impella was carefully pulled out from the aorta (Fig. 5).

**Fig. 6.**
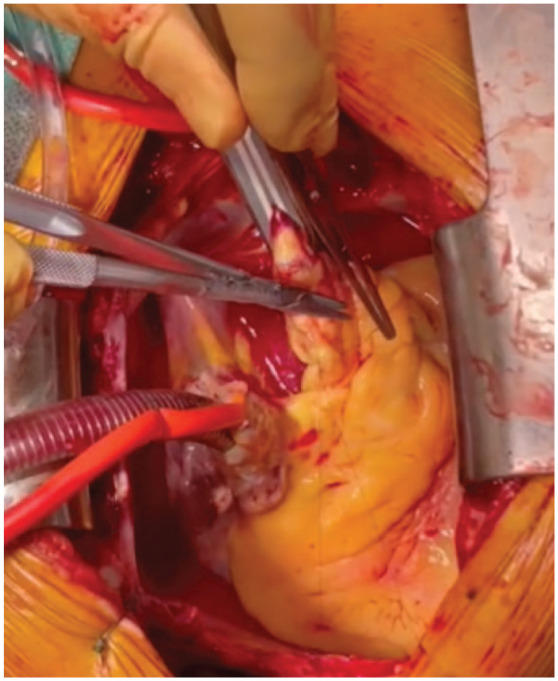
Temporary closure of aortotomy site and cross-clamp removal.

**Fig. 7.**
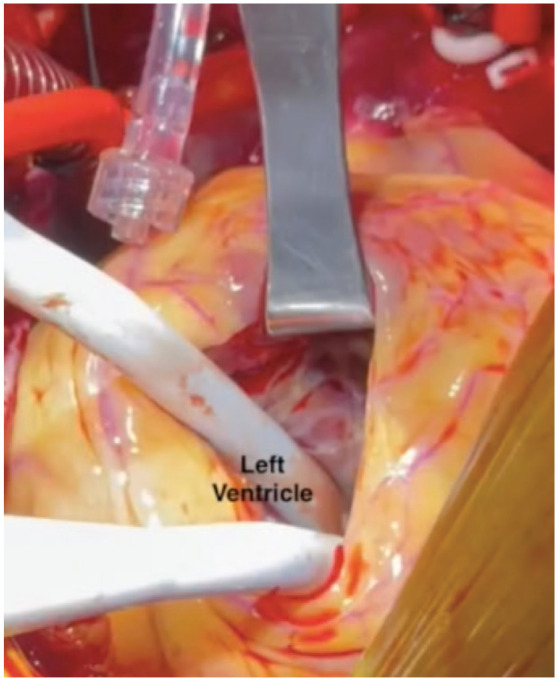
Left ventricular endocardial examination for residual clot through ventriculostomy.

The LV was inspected through the left ventriculotomy and freed from residual clots. The aortotomy was temporarily closed using a nonabsorbable monofilament suture in a running fashion (Fig. 6). After making sure no thrombi were seen in the LV cavity, the cross-clamp was released (Fig. 7); the rest of the operation was done on a beating heart to minimize the total ischemic time and right ventricular dysfunction.

**Fig. 8.**
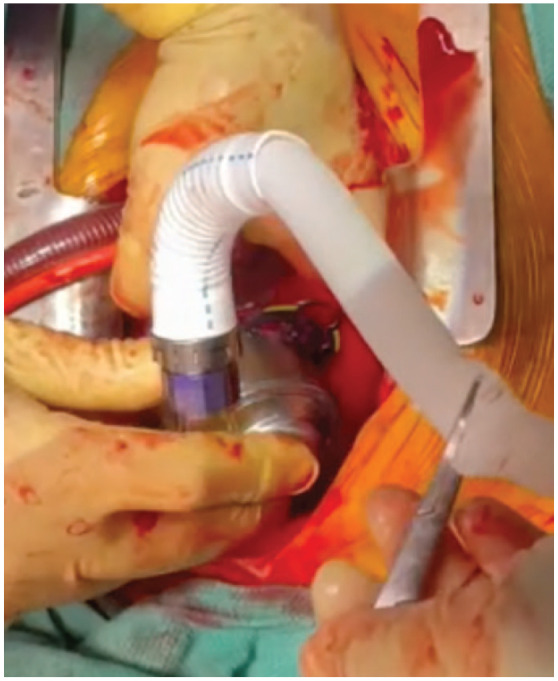
Placement of the sewing ring and inflow portion.

The sewing ring was sutured to the apex of the LV, the pump was secured to the sewing ring (Fig. 8), and the LV was de-aired in standard fashion.^7^ The side-biting clamp was placed under the previously sutured aortotomy. The suture was removed, and an anastomosis to the outflow graft was completed using a running Prolene suture. The rest of the operation was performed in standard fashion.

## Discussion

Intraoperative TEE or transthoracic echocardiogram are valuable tools for detecting LV and Impella inflow thrombi during durable LVAD implantation.^8^ When an LV or inflow site thrombus is visualized before the insertion of a durable LVAD, the evacuation of the entire clot should be performed via a ventriculotomy either with a beating or arrested heart.^7,9^

In patients with Impella 5.5 support as a bridge to durable LVAD, the device is usually stopped before CPB initiation, and the device is retracted into the insertion sheath. However, this sequence cannot be applied if there is a thrombus in the inflow portion of the device due to the risk of clot dislodgement and systemic embolization. Minimal manipulation of the heart before clot evacuation and early aortic cross-clamp application to decrease the chances of emboli showers are necessary. A padded aortic cross-clamp must be applied to avoid fracturing the shaft of the device and prevent aortic intimal injury. Some clinicians report not using an aortic cross-clamp, but we view using the cross-clamp in the presence of a thrombus located on the left side chambers (even when attached to a device) as a safety measure to minimize the risk for systemic embolization. Although not considered at the time, we recognize an alternative strategy is to remove the Impella through the ventriculotomy to avoid passing the clot through the aortic valve and detaching into the ventricle.

Right ventricular dysfunction is associated with prolonged aortic cross-clamp time; therefore, the aortic cross-clamp should be released as soon as the inflow site thrombus and Impella device have been removed.^10^ Thus, closing the aortotomy after Impella explantation to reuse it later will allow for cross-clamp removal and avoid major bleeding. Coring the LV before suturing the sewing ring provides better visualization of the ventricle and enables assessment for additional thrombi. Once the area is cleared of thrombi, the cross-clamp is released. The outflow graft is appropriately trimmed. The side-bite aortic clamp is placed under the temporarily closed aortotomy so that an end-to-side anastomosis between the outflow graft and the ascending aorta can be completed.

## Conclusions

With the increasing use of Impella devices, our technique highlights a practical solution to minimize the risk of embolization in patients with an LV or inflow site thrombus at the time of switching from Impella to durable LVAD support.

## References

[1] Rihal CS Naidu SS Givertz MM , et al. 2015 SCAI/ACC/HFSA/STS clinical expert consensus statement on the use of percutaneous mechanical circulatory support devices in cardiovascular care: endorsed by the American Heart Assocation, the Cardiological Society of India, and Sociedad Latino America. J Am Coll Cardiol 2015; 65: e7–e26. 10.1016/j.jacc.2015.03.036 25861963

[2] Roka-Moiia Y Li M Ivich A , et al. Impella 5.5 versus Centrimag: a head-to-head comparison of device hemocompatibility. ASAIO J 2020; 66: 1142–1151. 33136602 10.1097/MAT.0000000000001283 PMC7594535

[3] Imaoka S Kainuma S Toda K , et al. Impella support as a bridge to surgery for severe mitral regurgitation with cardiogenic shock. Circ Rep 2021; 3: 178–181. 33738351 10.1253/circrep.CR-21-0016 PMC7956879

[4] Lamarche Y Cheung A Ignaszewski A , et al. Comparative outcomes in cardiogenic shock patients managed with Impella microaxial pump or extracorporeal life support. J Thorac Cardiovasc Surg 2011; 142: 60–65. 20880553 10.1016/j.jtcvs.2010.07.075

[5] Nguyen D Ellison D Ngo C , et al. Intraventricular free-floating thrombus in an Impella-supported patient: damage control in a no-win scenario. JACC Case Rep 2020; 2: 886–888. 34317374 10.1016/j.jaccas.2020.04.045 PMC8302019

[bibr6-15569845231196862] Asadourian M Sharma AV Kiel R , et al. Removal of Impella in the setting of left ventricular thrombus: a potential indication for cerebral embolic protection devices. VAD J 2021; 7: e2021714.

[7] Salas De Armas IA Patel JA Akay MH , et al. Off-pump continuous-flow left ventricular assist device implantation. Texas Hear Inst J 2021; 48: 8–11. 10.14503/THIJ-19-7033 PMC8108714 33946106

[8] Gregoric ID Poredos P Jezovnik MK , et al. Use of transthoracic echocardiogram to detect left ventricular thrombi. Ann Thorac Surg 2021; 111: 556–560. 32687826 10.1016/j.athoracsur.2020.05.106

[9] Whitson BA . Surgical implant techniques of left ventricular assist devices: an overview of acute and durable devices. J Thorac Dis 2015; 7: 2097–2101. 26793329 10.3978/j.issn.2072-1439.2015.11.53 PMC4703668

[10] Levin A . The cardiovascular effects of aortic clamping and unclamping. South African J Anaesth Analg 2010; 16: 62–71.

